# Milk Products Containing Bioactive Tripeptides Have an Antihypertensive Effect in Double Transgenic Rats (dTGR) Harbouring Human Renin and Human Angiotensinogen Genes

**DOI:** 10.1155/2010/287030

**Published:** 2009-11-30

**Authors:** Tiina Jauhiainen, Taru Pilvi, Zhong Jian Cheng, Hannu Kautiainen, Dominik N. Müller, Heikki Vapaatalo, Riitta Korpela, Eero Mervaala

**Affiliations:** ^1^Institute of Biomedicine, Pharmacology, University of Helsinki, 00014 Helsinki, Finland; ^2^Research Center, Valio Ltd, 00370 Helsinki, Finland; ^3^Medcare Oy, 44100 Äänekoski, Finland; ^4^Max Delbruck Center for Molecular Medicine, 13125 Berlin, Germany; ^5^Foundation for Nutrition Research, 00039 Helsinki, Finland

## Abstract

Tripeptides isoleucyl-prolyl-proline (IPP) and valyl-prolyl-proline (VPP) act as ACE inhibitors in vitro. Double transgenic rats (dTGR) harbouring human renin and human angiotensinogen genes develop malignant hypertension due to increased angiotensin II formation. The present study was aimed to evaluate possible antihypertensive effect of IPP and VPP in this severe model. Four-week-old dTGR were randomized in three groups to receive: (1) water (control), (2) fermented milk containing IPP and VPP, and (3) IPP and VPP dissolved in water for three weeks. Fermented milk, but not peptides in water, attenuated the development of hypertension in dTGR by 19 mmHg versus the control group (*P* = .023). In vitro vascular function tests showed that high concentrations of the peptides evinced ACE inhibitory properties. In other hypertension related variables, no significant differences between the treatment groups were found. In conclusion, fermented milk product containing IPP and VPP prevents development of malignant hypertension in an animal model.

## 1. Introduction

Fermented milk products containing biologically active tripeptides, isoleucyl-prolyl-proline (IPP) and valyl-prolyl-proline (VPP), have been shown to attenuate the development of hypertension in spontaneously hypertensive rats (SHRs) [1–4] and to reduce blood pressure in mildly hypertensive subjects [5–10]. These antihypertensive peptides have been reviewed in detail recently [11–14]. The present hypothesis as to the antihypertensive effect is that of angiotensin-converting enzyme (ACE) inhibition, which has been shown both in vitro and in vivo [1, 3, 15, 16]. In addition, fermented milk containing IPP and VPP lowers ACE activity in the aorta [17] and elevates plasma renin activity (PRA) in a long-term treatment of SHRs [3]. Furthermore, these peptides protect endothelial function in vitro during a 24-hour incubation [18].

Despite these findings, the exact antihypertensive mechanisms behind IPP and VPP have not been confirmed. As described previously, double transgenic rats (dTGR) harbouring human renin and human angiotensinogen genes develop malignant hypertension, cardiac hypertrophy, renal damage, and endothelial dysfunction due to increased angiotensin II (Ang II) formation by the transfected human genes [19–21]. Inhibitors of ACE and renin, and AT_1_ receptor antagonists have been shown to reduce blood pressure and vascular inflammatory responses, decrease adhesion molecule expression in the kidneys, and improve endothelial function in dTGR [22]. Thus, these animals are considered to be a sensitive model for studying agents which are thought to modify the function of the renin-angiotensin system (RAS).

The aim of the present study was to evaluate the possible antihypertensive effect of fermented milk preparation containing IPP and VPP, and the pure peptides in drinking water, for the first time in this severe model of hypertension, dTGR, which overexpresses RAS. The role of ACE inhibitory activity of IPP and VPP, and their effects on hypertension related biochemical markers, were also studied.

## 2. Material and Methods

### 2.1. Animals and Diets

The study protocol was approved by the Animal Experimentation Committee of the Institute of Biomedicine, Helsinki, Finland. Four-week-old body-weight-matched (61 ± 2.2 g) dTGRs harbouring human renin and human angiotensinogen genes in a Sprague-Dawley (SD) background (Biotechnology and Animal Breeding Division, Füllinsgdorf, Switzerland) were used. The treatment groups (*n* = 15 in the control group and *n* = 10 in the fermented milk and peptide groups in the beginning of the study) were as follows:

1. *Control group:* dTGR + water as drinking fluid (*n* = 9 at the end of the treatment period),
2. *Fermented milk group:* dTGR + milk product containing IPP (1.8 mg/100 mL) and VPP (1.8 mg/100 mL) (Valio Ltd, Helsinki, Finland) (*n* = 6 at the end of the treatment period),
3. *Peptide group:* dTGR + IPP (3.4 mg/100 mL) and VPP (3.4 mg/100 mL) (IPP and VPP, Bachem AG, Bubendorf, Switzerland) in water as drinking fluid (*n* = 8 at the end of the treatment period).

The concentration of the peptides in the drinking fluid was based on our previous experience on the fluid consumption of spontaneously hypertensive rats in similar experiments (2–4). The animals had free access to feed (2018, Harlan Nederland, Horst, The Netherlands) and drinking fluid during the whole 3-week treatment period. Normotensive Sprague-Dawley rats of the same age (water as drinking fluid) served as healthy controls to see the age related changes in blood pressure. 

### 2.2. Blood Pressure Measurement

Systolic blood pressure was measured by using a tail-cuff blood pressure analyser (IITC Life Science, Woodland Hills, CA, USA). Before measurement, the rats were kept at +32°C for 15 minutes to improve the detection of the pulse of the tail artery. The systolic blood pressure value was defined as a mean of three successful measurements without disturbances of the signal. 

### 2.3. Fluid and Feed Consumption

Fluid consumption was monitored daily and feed consumption weekly. The energy nutrient and mineral contents in the fermented milk, as well as the IPP and VPP contents to control their preservation, were analysed by the peptide research group in Valio Ltd (Helsinki, Finland). The tripeptide content of *Lactobacillus helveticus* fermented milk was determined by the modified method of Masuda et al. [23], by collecting the peptide fraction by gel filtration chromatography (Superdex Peptide HR 10/30 Amersham Pharmacia Biotech, Bucks, UK) and analysing it by reversed phase HPLC (Waters Alliance HPLC system, Milford, MA, USA), using synthetic peptides as standards. 

### 2.4. In Vitro ACE Inhibitory Activity

The mesenteric artery was cleaned of adherent connective tissue. Three-mm-long arterial rings were placed between stainless steel hooks and suspended in an organ bath chamber in Krebs-Ringer buffer (pH 7.4) of the following compositions (mM): NaCl 119.0, NaHCO_3_ 25.0, glucose 11.1, CaCl_2_ 1.6, KCl 4.7, KH_2_PO_4_ 1.2, and MgSO_4_ 1.2, and aerated with 96%  O_2_ and 4%  CO_2_. The rings were equilibrated for 45 minutes at +37°C with a resting tension of 1.0 g. The presence of the functional endothelium of the preparations was confirmed by observing an endothelium-dependent relaxation response to the 1 *μ*M acetylcholine (ACh) in the 1 *μ*M noradrenaline (NA) precontracted arterial rings. The ACE inhibitory activity of IPP and VPP in vitro was determined by preincubating the arterial rings from normotensive Sprague-Dawley rats with tripeptides for 10 minutes and measuring the response to a single administration of angiotensin I (1 *μ*M) or angiotensin II (1 *μ*M). 

### 2.5. Urine, Plasma, and Tissue Samples

During weeks 1 and 3 of the treatment period, the rats were housed individually in metabolic cages for 24 hours. The consumption of drinking fluids and feed was measured and the intake of energy nutrients, minerals, and IPP and VPP was calculated during the 24-hour period (Table 1). Urine volumes were measured and samples were stored at −80°C for microalbumin measurements. 

At the end of the study, the rats were rendered unconscious with CO_2_/O_2_ (95%/5%) (AGA, Riihimäki, Finland) and decapitated. The blood samples were put into chilled glass tubes with EDTA as an anticoagulant. The blood samples were centrifuged (1800 ×g, 20 minutes, +4°C), and the plasma separated and stored at −80°C until analysed. The heart and kidneys were removed, washed with saline, blotted dry and weighed, and the ratios to body weight were calculated. 

### 2.6. Biochemical Determinations

Urinary albumin excretion was measured by ELISA, using rat albumin as a standard (Celltrend GmbH, Luckenwalde, Germany). Plasma renin activity (PRA) was measured using a radioimmunoassay of angiotensin I (Medix Angiotensin I test, Medix Biochemica, Kauniainen, Finland); aldosterone was measured by radioimmunoassay (Diagnostic Products Co, Los Angeles, CA, USA); and plasma B-type natriuretic peptide (BNP) was measured by radioimmunoassay as described in detail earlier [24]. 

### 2.7. Renal Morphology

For morphological studies, the renal samples were fixed in 4% buffered paraformaldehyde at room temperature, dehydrated in graded alcohol, and embedded in paraffin. Two-to-three-*μ*m sections were cut with a microtome and placed on glass. The sections were deparaffinised and rehydrated before staining with Masson's trichrome. Renal damage was analysed in a blinded fashion and divided into four scores of 0–4 (0 = normal and 4 = severe damage) as described previously [25]. 

### 2.8. Statistics

The results are expressed as means with standard errors (SE). Statistically significant differences between the groups were tested by permutation type ANOVA with Hommel's adjustment if appropriate. Permutation type test was used because there are a small number of observations in each group and assumptions underlying to corresponding parametric test cannot be relied on.

## 3. Results

### 3.1. Body Weight and Blood Pressure

**Figure 1:**
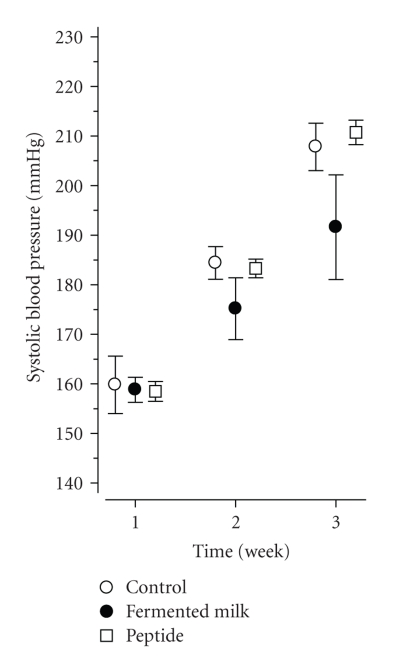
The mean (±SE) systolic blood pressure in different treatment groups. The number of animals in the beginning of the experiment: control 15, fermented milk and peptide groups 10/group. Week 3: 9, 6 and 8, respectively, (*P* = .023 between the groups).

Body weight gain development was similar between the groups during the 3-week treatment (Table 1). Already in the beginning of the study, 4-week-old dTGRs were clearly hypertensive, with systolic blood pressure about 160 mmHg (Figure 1). The development of severe hypertension was fast, about 25 mmHg in a week in the dTGR control and peptide groups, but less in the fermented milk group, about 15 mmHg in a week (Figure 1). At the end of the treatment period, the mean blood pressure was 192 ± 4 mmHg in the control group, 174 ± 5 mmHg in the fermented milk group, and 189 ± 4 in the peptide group (Figure 1) (*P* = .023 between the groups). The blood pressure of the normotensive SD rats remained at the same level during the whole study period (132 ± 2 mmHg in the beginning and 128 ± 1 mmHg in the end of the study, not shown in the figure). 

During the treatment period, six rats from the controls, two rats from the peptide group, and four rats from the fermented milk group died during nights and therefore could not be autopsied. 

### 3.2. Fluid and Feed Consumption

In daily measurements of the fluid intake, there were no differences between the groups and also the energy intake was similar between the groups (data not shown). This explains the similar body weight at the end of the experiment. The mean estimated daily peptide intake (IPP+VPP) during the whole study period was 5.4 mg/kg in the fermented milk group and 10.9 mg/kg in the peptide group. However, the 24-hour monitoring in metabolic cages, showed differences in the feed and fluid intake being lowest in the fermented milk group (Table 1). Therefore, calcium, magnesium, and sodium intake was also lowest in the fermented milk group, respectively. Based on these data, the peptide intake (IPP+VPP) during the 24-hour monitoring was 4.1 ± 1.1 mg/kg body weight in the fermented milk group and 14.1 ± 1.4 mg/kg in the peptide group. These contradictory findings in metabolic caging may be due to too short period for the animals to become acclimatized in a new environment. Therefore, the repeated daily recordings can be regarded more reliable.

### 3.3. In Vitro Bioassay of ACE Inhibitory Activity

To test the ACE inhibitory activity of IPP and VPP in the in vitro bioassay, mesenteric artery rings of normotensive SD rats were used. IPP and VPP (0.33 mM) administered together inhibited 84% of the angiotensin I (1 *μ*M)-induced contraction, but did not reduce angiotensin II-induced contraction.

### 3.4. Cardiac and Renal Hypertrophy

The dTGRs showed marked cardiac and renal hypertrophy compared to the normotensive SD controls. Neither fermented milk nor peptides affected the heart or kidney weight to body weight ratios (Table 2). 

### 3.5. Renal Morphology

The arteriales of untreated dTGRs showed perivascular inflammation and concentric hypertrophy with medial and intimal proliferations. The glomeruli had notable mesangial expansion. The tubules were only minimally affected.

The damage score was 3.3 ± 0.2 in the dTGR control group, 3.0 ± 0.3 in the fermented milk group, and 2.8 ± 0.2 in the peptide group (*P* = .53).

### 3.6. Biochemical Determinations

During the treatment period, all the dTGR groups developed severe albuminuria, compared to the healthy SD controls (*P* < .05), as a marker of renal damage according to the renal damage score. At the end of the treatment period, there was no difference in albumin excretion between the three treatment groups (Table 2). 

Aldosterone values tended to be highest in the fermented milk group. However, the difference did not reach statistical significance obviously due to large variations. There was no statistical difference between the groups in PRA or BNP (Table 2). 

## 4. Discussion

The present study with double transgenic rats harbouring human renin and angiotensinogen genes aimed to evaluate the blood-pressure-lowering effect and its mechanisms of the two casein-derived tripeptides IPP and VPP in a fermented milk product. According to previous studies by us [2–4] and others [1] using spontaneously hypertensive rats, the present study shows for the first time that milk-based products containing these peptides act antihypertensively even in a malignant form of experimental hypertension. ACE inhibitory activity of the pure peptides was confirmed in vitro, but the effect could not been seen in vivo despite prevention of hypertension*.*

The difference from the control group was of the same magnitude (about 20 mmHg) as in our previous studies on SHRs [2–4]. The attenuation in blood pressure development was not seen in the peptide group, despite the higher intake of peptides compared to the fermented milk group. Thus the antihypertensive effect of fermented milk cannot be explained alone by the ACE inhibitory activity of IPP and VPP, contrary to the earlier findings on a less severe hypertension model, SHR [3]. The antihypertensive effect of fermented milk may also be related to other components such as favourable milk mineral contents, more calcium and potassium, and less sodium, as suggested and shown by us recently [4]. The bioavailability of the peptides versus peptides in water may be improved by other milk components. Furthermore, caseinophosphopeptides are known to increase the absorption of calcium [26, 27], a blood pressure lowering electrolyte [28, 29]. The antihypertensive effect of fermented milk might also relate to other effects of milk-derived peptides [30] or other antihypertensive mechanisms related to the RAS or L-type calcium channels as described recently on some synthetic dipeptides [31].

One explanation for the fairly weak antihypertensive and lack of biochemical effects in the fermented milk group and the lack of effect in the peptide group may be the short treatment period, low dose of peptides, and the fast development of severe hypertension with death of the animals. In our earlier experimental [2–4] and clinical studies [6, 7], the antihypertensive effect was first seen after five or six weeks of treatment, which was not possible in the present study. 

The intake of tripeptides in the fermented milk group and the peptide group remained lower than in our earlier studies [2–4]. The present doses may not have been sufficient to antagonise the severe hypertension of dTGRs caused by an over-expressed RAS, although ACE inhibition has been shown in SHRs in vitro and in vivo [2–4]. In our previous studies [2, 4], peptides in water have also been less effective than the fermented milk product in lowering blood pressure possibly due to their weaker bioavailability from water. Only partial intestinal absorption of the unmetabolized di- or tripeptides has been demonstrated in the rat [32], in the pig [33], and in man [34, 35].

Biochemical determinations did not show clear differences between the groups in any of the markers. With this rat model, therefore, ACE inhibition does not seem to explain the blood pressure lowering effect in the fermented milk group. According to a previous study [36], the plasma concentration of BNP was elevated in dTGRs related to cardiac pressure. The peptide treatments in this study were not able to antagonise clearly this increase. It may be that this ineffectiveness can be explained by the lack of clear cardiac hypertrophy in the present study. However, the slightly lower BNP value in the fermented milk group could indicate lower risk for cardiac hypertrophy, if it were possible to continue the experiment longer. 

There are two major concerns in the study. Firstly, due to fast development of severe hypertension, part of the animals, mainly from the untreated (control) group, died during the intervention thus weakening the power of the study. Furthermore, from the same reason the intervention period remained shorter than that in our previous studies where the antihypertensive effect has been more evident [2–4]. Secondly, there is discrepancy between the continuous feed and drinking fluid recording and the data from 24-hour metabolic caging. The former seems to be more reliable, because the body weight development did not differ and daily observation of the animals did not indicate markers of poor physical condition.

## 5. Conclusions

The present study shows that the weak ACE inhibitory activity of IPP and VPP in drinking water is not able to prevent the severe hypertension in this rapidly developing hypertension model, although the antihypertensive effect is evident in SHRs. However, the peptides in fermented milk clearly antagonized the increase in blood pressure suggesting improved kinetics of the peptides in this product or other active components present. 

## Figures and Tables

**Table 1:** Body weight, urine volume, and the intake of feed, fluids and minerals during the 24-hour period in metabolic cages at the end of the three-week treatment period (mean ± SE, *n* = 6–9).

Variable	Control	Fermented milk	Peptide	Difference between the groups, *P*
Body weight, g	235 ± 13	221 ± 11	239 ± 7	.53
Urine volume, mL	33 ± 3	33 ± 4	44 ± 4	.085
Feed intake, g	26 ± 1	16 ± 1	26 ± 0.5	<.001
Fluid intake, mL	37 ± 4	24 ± 8	47 ± 4	.032
Energy, kJ	352 ± 15	285 ± 27	353 ± 7	.014
Sodium, mg	59 ±2	44 ± 3	60 ± 1	<.001
Potassium, mg	175 ± 7	188 ± 28	175 ± 3	.76
Calcium, mg	260 ± 11	213 ± 21	261 ± 5	.025
Magnesium, mg	51 ± 2	36 ± 2	52 ± 1	<.001

**Table 2:** Biochemical variables and cardiac and renal hypertrophies at the end of the three-week treatment period (mean ± SE, *n* = 6–9).

Variable	Control	Fermented milk	Peptide	Difference between the groups, *P*
Urinary albumin, mg/d	22 ± 4	35 ± 8	30 ± 4	.20
PRA, ng Ang I/mL/h	90 ± 17	99 ± 30	95 ± 19	.98
BNP, pmol/L	3.1 ± 0.5	2.3 ± 0.2	3.5 ± 0.3	.24
Aldosterone, pg/mL	1228 ± 302	2551 ± 921	1494 ±218	.18
Cardiac hypertrophy index (mg/g)	5.3 ± 0	5.1 ± 0	5.1 ± 0	.59
Renal hypertrophy index (mg/g)	4.6 ± 0	4.8 ± 0	4.8 ± 0	.61
